# Longitudinal metabolomics study of phosphate‐adenine‐guanosine‐glucose‐saline‐mannitol stored red blood cells

**DOI:** 10.1111/trf.70070

**Published:** 2026-01-10

**Authors:** Gürkan Bal, Maia Dzamashvili, Zhuoran Li, Magda Babina, Nidal Toman, Abdulgabar Salama

**Affiliations:** ^1^ Institute of Allergology Charité – Universitätsmedizin Berlin, Corporate Member of Freie Universität Berlin and Humboldt‐Universität zu Berlin Berlin Germany; ^2^ Fraunhofer Institute for Translational Medicine and Pharmacology ITMP Immunology and Allergology Berlin Germany; ^3^ Klinikum Oldenburg Universitätsklinik für Innere Medizin ‐ Hämatologie und Onkologie Oldenburg Germany; ^4^ Department of Psychology Touro University Berlin Berlin Germany; ^5^ Department of Gynecology, Charité‐Universitätsmedizin Berlin, corporate member of Freie Universität Berlin Humboldt‐Universität zu Berlin and Berlin Institute of Health Berlin Germany

**Keywords:** additive solution, mast cells, metabolomics, RBC, storage lesion

## Abstract

**Background:**

The storage of red blood cells (RBCs) is essential for transfusion but leads to storage lesions that compromise RBC quality and increase the risk of transfusion‐related adverse effects, including allergic transfusion reactions (ATRs). Understanding storage‐induced metabolic change is crucial for enhancing transfusion safety.

**Study Design and Methods:**

We conducted targeted metabolomic profiling of RBC supernatants stored in PAGGS‐M over 42 days, collecting 161 weekly samples from 23 units. We analyzed 188 metabolites across six compound classes (hexoses, amino acids, biogenic amines, acylcarnitines, glycerophospholipids, and sphingolipids) along with 20 hematological parameters. Additionally, a mast cell degranulation assay evaluated the impact of these changes on ATR risk.

**Results:**

Over 100 of the 188 metabolites changed significantly during storage, indicating diverse pathway alterations. Key findings include the accumulation of acylcarnitines and depletion of methionine, with mast cell degranulation significantly increased in supernatants from 42‐day‐old pRBCs versus fresh units. Notably, methionine depletion coincided with a critical transition phase in storage‐associated metabolic aging. In addition, elevated levels of acylcarnitines correlated with increased markers of membrane damage, highlighting potential mechanisms underlying allergic transfusion reactions.

**Discussion:**

This study highlights the impact of storage‐induced metabolic changes in exacerbating transfusion‐related complications. Therefore, optimization of additive solutions, such as stabilizing methionine levels during storage, may mitigate storage lesions and improve outcomes. Additionally, the storage‐time‐related increase in mast cell activation suggests a need for targeted interventions, particularly for patients vulnerable to ATRs.

**Abbreviations**
ATR: allergic transfusion reaction
CPD: citrate‐phosphate‐dextrose
EVs: extracellular vesicles
extHb: extracellular hemoglobin
MCs: mast cells
MEBA: Multivariate Empirical Bayes Analysis
PAGGS‐M: phosphate–adenine–guanosine–glucose–saline–mannitol
PCA: principal component analysis
pRBCs: packed red blood cells
PTR: post‐transfusion recovery
QM: quality marker
RBCs: red blood cells
RDW‐SD: red cell distribution width – standard deviation
RF: random forest
SAM: S‐adenosylmethionine

## 1 INTRODUCTION

Red blood cells (RBCs) are the primary cellular component of human blood. Mature RBCs are unique because they lack a nucleus and most organelles, such as mitochondria, ribosomes, the endoplasmic reticulum, and the Golgi apparatus.^1^ Consequently, they cannot perform aerobic metabolism. Instead, RBC metabolism relies exclusively on anaerobic glycolysis and the pentose phosphate pathway to produce essential metabolic intermediates, including ATP and NADPH. This metabolic strategy sustains them for an average lifespan of 115 days in circulation.^2^ During gas exchange at the peripheral tissue level, RBCs are constantly exposed to reactive oxygen species. This leads to progressive changes, collectively known as “RBC senescence,” which includes reduced membrane deformability and metabolic imbalances.^3^ Because RBCs cannot synthesize new enzymes to neutralize cellular waste or regenerate membrane components, their half‐life shortens, ultimately resulting in macrophage‐mediated clearance from circulation.^4^

Packed red blood cells (pRBCs) are a commonly used blood component for life‐saving therapeutic intervention in anemic, surgical, oncologic, and trauma patients. They are routinely prepared from 450 to 500 mL of whole blood collected in citrate‐phosphate‐dextrose (CPD) anticoagulant, which is subsequently processed through centrifugation and filtration to separate components and achieve leukodepletion. The processed RBC units contain ~110 mL additive solution and 5–10 mL of residual plasma, and are stored under refrigerated conditions (4 ± 2°C) for a maximum of 35–49 days.

Under ex vivo storage conditions, pRBCs undergo accelerated senescence compared to circulating RBCs in vivo, leading to a well‐characterized phenomenon known as “storage lesions.”^5^ Storage lesions encompass a range of physical, chemical, and metabolic changes, including impaired energy metabolism, decreased antioxidant defense capacity, disrupted membrane lipid asymmetry, increased membrane rigidity, and reduced RBC deformability.^6^, ^7^ These alterations diminish the RBCs' oxygen‐carrying capacity and accelerate clearance; ~25% of transfused RBCs are cleared within the first 24 h, and this fraction increases with storage time.^8^ Critically, storage lesions contribute significantly to transfusion‐associated complications such as transfusion‐related acute lung injury, hypercalcemia, thrombophlebitis, citric acid toxicity, and transfusion‐related immunomodulatory effects.^9^, ^10^, ^11^

Previous metabolomics studies have greatly enhanced our understanding of the biochemical pathways involved in RBC aging and storage‐related alterations^12^, ^13^, ^14^, ^15^, ^16^, ^17^ and have guided improvements in storage practices.^18^ Nevertheless, the challenge of storage lesions remains unresolved in clinical settings. Since earlier investigations have not fully resolved this problem, we expand the metabolic scope by simultaneously quantifying six key metabolite classes: hexoses, amino acids, biogenic amines, acylcarnitines, glycerophospholipids, and sphingolipids using a targeted metabolomics approach with absolute quantification. To our knowledge, this specific combination has not been quantified in a single targeted assay. We focused on the supernatant because the metabolic profile of the extracellular medium serves as a functional reflection of intracellular RBC metabolism. Due to membrane transport mechanisms and equilibration, metabolites and waste products are continuously exchanged, making the supernatant a proxy for the internal metabolic state of the erythrocyte. This analysis enabled precise characterization of biochemical changes and identification of specific biomarkers associated with storage lesions.

## 2 STUDY DESIGN AND METHODS

### 2.1 Study design

Briefly, using triple quadrupole mass spectrometry, 188 metabolites from 6 compound classes (hexoses, amino acids, biogenic amines, acylcarnitines, glycerophospholipids, and sphingolipids) were quantified using a targeted quantitative metabolomics workflow. Bioinformatics algorithms were applied to the quantitative metabolomics dataset to identify an optimized subset of biomarkers which may be used to manipulate RBC metabolism during cold storage period. Quality Marker (QM) parameters: hemogram and extracellular hemoglobin levels (extHb) were assessed throughout storage to monitor RBC integrity and serve as complementary indicators of storage lesion severity, facilitating interpretation of concurrent metabolic alterations. In addition to metabolomics, we performed a mast cell degranulation assay to determine whether storage‐induced changes in pRBC supernatants translate into measurable biological activity relevant to transfusion‐related reactions.

### 2.2 Donor cohort

The donor cohort consisted of 23 healthy volunteers under the age of 40. To ensure a balanced representation, donors were selected based on sex (12 female, 11 male), Rh factor (13 Rh‐positive, 10 Rh‐negative), and all four ABO blood groups. Although these demographic criteria were used to assemble a balanced cohort, all pRBC units were anonymized before analysis. As a result, individual demographic data were not linked to specific samples, and analyses were conducted using anonymized samples.

### 2.3 Sample collection and storage

Whole blood was collected according to the German National Blood Center guidelines (Blood donation services‐ZTB, Berlin). We used DQE 7241LC blood bags from Maco Pharma International GmbH, which contained 70 mL CPD in whole blood bag and 110 mL PAGGS‐M (phosphate‐adenine‐guanosine‐glucose‐saline‐mannitol) in RBC bag. All whole blood units were processed to obtain 23 pRBC units. All equivalent RBC products were stored for up to 42 days under standard conditions (4 ± 2°C). A 10 mL aliquot was aseptically drawn into satellite bags at 0, 7, 14, 21, 28, 35, and 42 days. Samples were centrifuged at 1800 rpm for 10 min (no brake); supernatants (750 μL) were transferred to 1.5 mL tubes and re‐centrifuged at 13,000 rpm for 5 min to remove residual cells. It should be acknowledged that although the centrifugation protocol efficiently removes cells and cellular debris, it does not eliminate small extracellular vesicles (EVs). Consequently, the lipid species identified in this study likely reflect a mixture of free lipids and EV‐associated lipids. Aliquots were frozen at −80°C for metabolomics; remaining material was used immediately. At the end of storage, all RBC units were checked for bacterial contamination. None of the RBC units were contaminated.

### 2.4 Quality marker (QM) measurements

Hemogram analysis was conducted using a Sysmex XE Sysmex 5000 hematology analyzer on small sample tubes containing a 1:10 dilution of whole blood. Results were subsequently multiplied by the dilution factor to obtain absolute cell counts. For extHb/hemolysis rate, 20 μL of supernatant of each sample was used to measure the extHb level in the supernatant with Plasma/Low Hb Analyzer (HemoCue).

### 2.5 Quantitative metabolomics framework

AbsoluteIDQ‐p180 kit (Biocrates Life Science AG, Innsbruck, Austria) was applied for analysis of pRBC supernatants on a triple‐quadrupole mass spectrometer (API4000, Sciex, Framingham, MA) with an electrospray‐ionization ion source coupled to a high‐performance liquid chromatography system (SIL‐HTc, Shimadzu, Japan). This framework enabled detection of (1) 21 amino acids and 21 biogenic amines by liquid chromatography separation followed by targeted tandem mass spectrometry (LC‐MS/MS) and (2) 91 glycerophospholipids (phosphatidyl‐ and lysophosphatidylcholines) and isomers, 40 acylcarnitines, 15 sphingolipids (sphingo‐ and hydroxysphingomyelins) and isomers, and the sum of hexoses by direct infusion MS/MS (flow injection analysis, FIA‐MS/MS). All assays were performed according to the manufacturer's recommendations (user manual UM_p180_ABSciex_11 and application note 1003‐1, Biocrates Life Science AG, Innsbruck, Austria). In brief, the manufacturer's internal standard solution and calibrator/quality control samples or 30 μL of supernatants were loaded onto a filter plate, derivatized using 5% phenylisothiocyanate in ethanol/water/pyridine (1/1/1, v/v/v), and extracted using 5 mM ammonium acetate in methanol. Remaining extracts were diluted and measured according to the manufacturer's instructions.

### 2.6 Statistics and data analysis

Peak integration and calculation of metabolite concentrations were performed with the Analyst (version 1.5.2, Sciex, Framingham, MA) and MetIDQ software (Biocrates Life Science AG, Innsbruck, Austria). Metabolite concentrations (μM) obtained from the primary analysis were uploaded to MetaboAnalyst for an in‐depth secondary evaluation. Within the platform, statistical modeling techniques, specifically Principal Component Analysis (PCA), Random Forest (RF), and Multivariate Empirical Bayes Analysis (MEBA), were employed to reveal significant metabolic alterations and identify potential biomarkers.^19^

### 2.7 Mast cell isolation and culture

To functionally contextualize the metabolomics findings, we tested whether supernatants from stored pRBCs trigger mast cell activation. Human skin mast cells (MCs) were isolated from foreskin biopsies obtained with written informed consent, as approved by the institutional ethics committee (protocol code EA1/204/10, 9 March 2018). Isolation followed established enzymatic digestion (collagenase, hyaluronidase, DNase I) and magnetic cell separation (anti‐human c‐Kit microbeads) protocols.^20^ MC purity (98%–100%) was confirmed by toluidine blue staining. Isolated MCs were then cultured for 3–4 weeks in basal Iscove's medium supplemented with stem cell factor and interleukin‐4.^21^ To assess the functional consequences of pRBC‐supernatant supplementation on mast‐cell degranulation, MC cultures were supplemented with 10% supernatant and incubated for 1 h before performing a β‐hexosaminidase assay.

### 2.8 Mast degranulation assay (β‐hexosaminidase assay)

We assessed β‐hexosaminidase release as a measure of mast cell degranulation induced by pRBC supernatants, serving as a model for transfusion‐related immediate adverse reactions. β‐hexosaminidase activity was assessed as previously described.^22^ Briefly, cells were pre‐incubated for 60 min in PAG‐CM buffer with pRBC supernatant and positive controls, including AER‐37 (IgER‐CL; IgE receptor cross‐linking‐mediated mast cell activation), Codeine (MRGPRX2‐mediated activation), and C48/80 (MRGPRX2‐mediated activation). After incubation, supernatants and cell lysates were treated with 4‐methylumbelliferyl‐N‐acetyl‐β‐D‐glucosaminide. The reaction was stopped, and fluorescence was measured to determine enzyme activity. The percentage of β‐hexosaminidase release was calculated and corrected for spontaneous release to obtain the net release.

## 3 RESULTS

### 3.1 Metabolomic changes in the supernatant of PAGGS‐M stored RBCs during a 42‐day storage period

**FIGURE 1:**
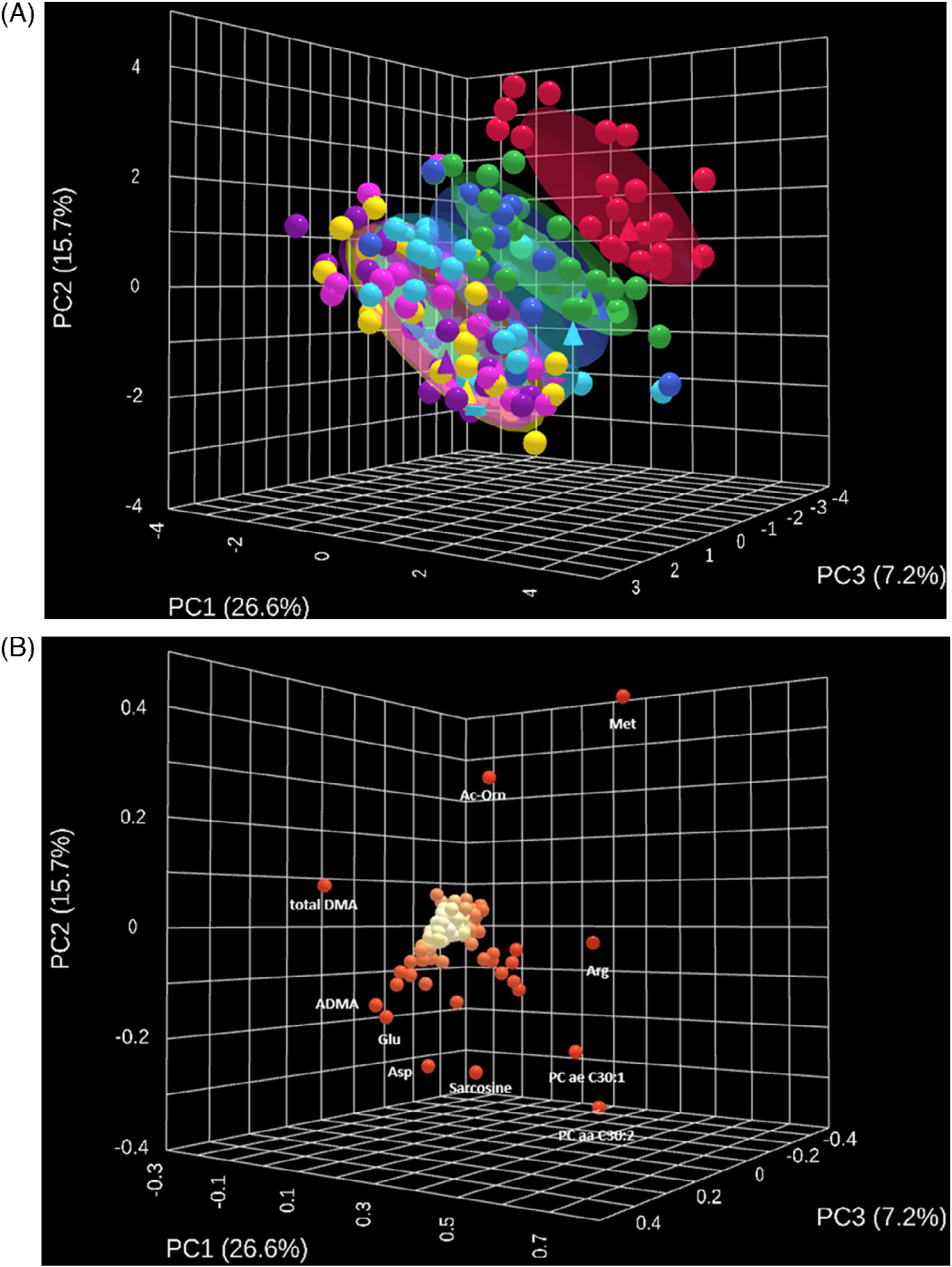
Principal component analysis (PCA) of RBC supernatant metabolomics across storage. (A) The 3D PCA score plot showing the clustering of samples based on their metabolite profiles. Each point represents one sample. Colors encode storage week (Week 0 red, Week 1 green, Week 2 blue, Week 3 cyan, Week 4 magenta, Week 5 yellow, Week 6 purple). Semi‐transparent ellipses indicate the 95% confidence region for each week. Explained variance: PC1 26.6%, PC2 15.7%, PC3 7.2%. (B) 3D PCA loading plot highlighting metabolites that contribute most to separation along PC1–PC3; top contributors are labeled. Point color encodes loading magnitude (lighter = lower, darker = higher). Ac‐Orn, acetyl‐ornithine; ADMA, asymmetric dimethylarginine; Arg, arginine; Asp, aspartate; DMA, dimethylamine; Glu, glutamate; Met, methionine.

A targeted metabolomic analysis was performed on supernatants of pRBCs using the Biocrates AbsoluteIDQ p180 kit. Samples were collected weekly over a 42‐day storage period from 23 healthy donors, resulting in 161 samples for analysis. In addition to quantifying 188 metabolites per sample (Data S1, worksheet I), extracellular hemoglobin levels (as part of QM parameters) and 20 hemogram variables were measured to ensure a comprehensive assessment of stored RBCs. PCA revealed distinct segregation of samples based on storage time along principal component 1 (PC1), which accounted for 26.6% of the total variance (Figure 1A). PC2 (15.7%) and PC3 (7.2%) appeared to suggest donor‐specific differences, such as sex and blood group, respectively. Samples from Week 0 clustered tightly together, while those from Weeks 1 and 2 formed a separate group. Samples from Weeks 3 to 6 clustered into another distinct group. The loading plot highlighted methionine, arginine, asparagine, PC ae C30:1, and PC aa C30:2 as the most influential metabolites driving the separation across principal components (Figure 1B).

**FIGURE 2:**
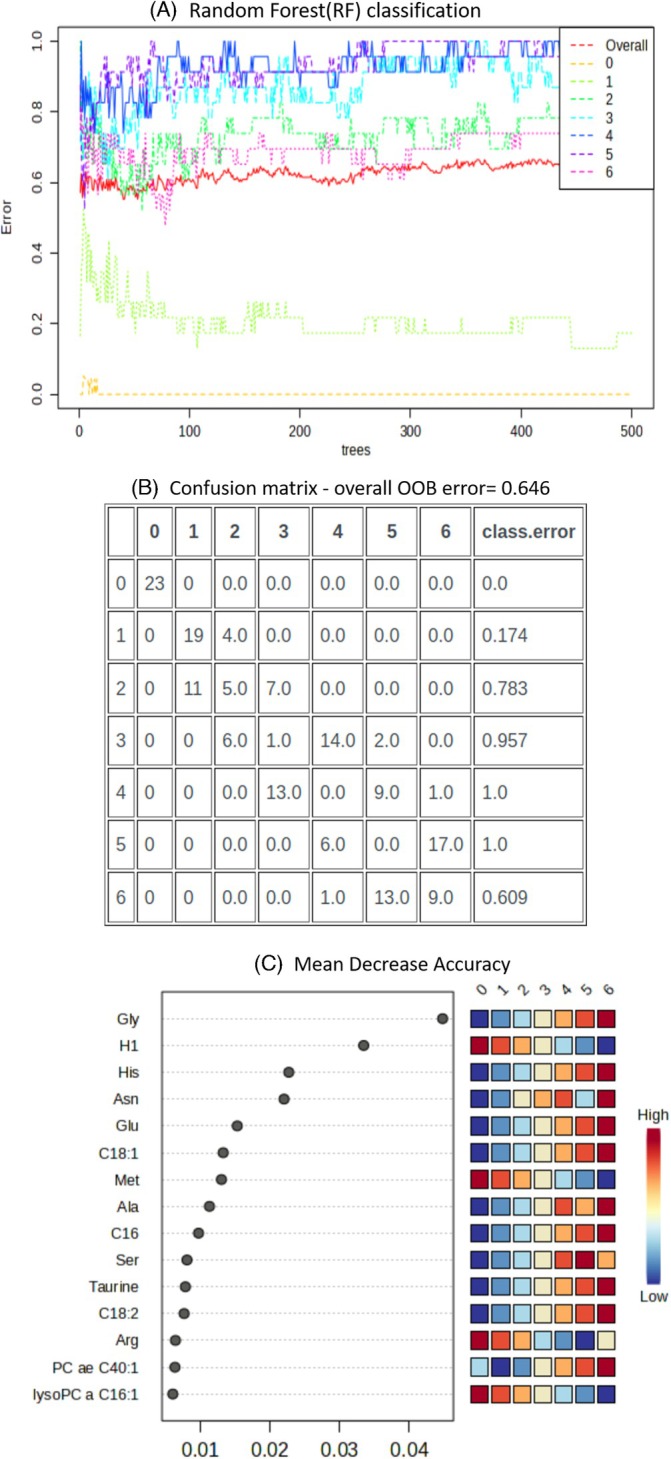
Random Forest model performance and feature importance analysis. (A) Random Forest (RF) Classification: The plot shows the error rate of the Random Forest classification model over the number of trees. Different colors represent the error rates for storage time classes. The overall error is also shown (red). (B) Confusion Matrix—Overall OOB Error = 0.646: The confusion matrix displays the out‐of‐bag (OOB) error rate for each class. The matrix provides detailed insights into the model's performance, indicating how many instances of each class were correctly or incorrectly classified. The class error rate for each class is also included. (C) Mean Decrease Accuracy: This plot ranks the features by their importance to the classification accuracy, measured as the mean decrease in accuracy. Higher values indicate more important features. The heatmap on the right shows the contribution of each feature across different classes, with colors indicating the level of contribution from low (blue) to high (red).

To model the relationship between storage time and metabolites, we conducted a Random Forest (RF) analysis. We trained the RF to predict storage week from metabolite profiles, with resampling stratified by donor ID to account for inter‐donor variability. This approach predicts storage time based on metabolite levels and identifies the most critical metabolites for prediction through variable importance scores. The RF model (Figure 2A), consistent with PCA results, achieved perfect classification accuracy for storage time at week 0 (OOB error = 0.0) and strong performance for week 1 (OOB error = 0.174). However, classification accuracy declined in subsequent weeks, leading to a suboptimal overall OOB error of 0.646 (Figure 2B). Despite these limitations, the variable importance matrix analysis revealed key metabolites—Glycine (Gly), Hexoses (H1), Histidine (His), Asparagine (Asn), Glutamic Acid (Glu), Octadecenoylcarnitine (C18:1), Methionine (Met), Alanine (Ala), Hexadecanoylcarnitine (C16), Serine (Ser), Taurine, Octadecadienylcarnitine (C18:2), and Arginine (Arg), whose concentrations varied significantly over the storage duration (Figure 2C, Data S1, worksheet II). To improve these findings and overcome the shortcomings of the RF analysis in classifying weeks 2–6, we complemented it with Multivariate Empirical Bayes (MEBA) time‐series analysis. MEBA is particularly suited for modeling dynamic temporal changes and provides a robust Bayesian framework for interpreting fluctuations in metabolite concentrations. As shown in Table 1, MEBA identified several metabolites exhibiting significant changes over the storage duration, as evidenced by their Hotelling's‐T2 statistics (Data S1, worksheet III). These key metabolites include Methionine (Met), Glycine (Gly), Octadecenoylcarnitine (C18:1), Octadecadienylcarnitine (C18:2), and Histidine (His), among others.

**TABLE 1:** Results of Multivariate Empirical Bayes (MEBA) time‐series analysis.

Metabolite	ID	Direction across storage	Approx. timing	Hotelling‐*T* ^2^	Biological relevance
Methionine	Met	↓	Early; near depletion by phase 2	7350.2	SAM donor for protein repair; depletion weakens proteostasis
Glycine	Gly	↑	Progressive	1641.2	Tracks GSH salvage and γ‐glutamyl cycling during oxidative stress
Octadecenoylcarnitine	C18:1	↑	Progressive	1264.6	Lipid peroxidation product; mast‐cell activating potential
Octadecadienoylcarnitine	C18:2	↑	Progressive	871.65	Tracks membrane remodeling and peroxidation; mast‐cell activating potential
Hexadecanoylcarnitine	C16	↑	Progressive	736.53	Accumulates with fatty‐acid release and β‐oxidation shunting
Histidine	His	↓/variable	Early–mid	739.61	Acid–base and antioxidant roles; consumption with oxidative stress
Hexoses	H1	↓	Early–mid	637.71	Glycolytic substrate consumption; energy decline with storage
Taurine	Taurine	↑	Progressive	618.03	Osmolyte and antioxidant support under stress
Alanine	Ala	↑	Progressive	542.68	Glycolytic by‐product; redox and nitrogen handling
Asparagine	Asn	↓	Progressive	509.36	Deamidation under oxidative stress; links to repair load
Serine	Ser	↓/variable	Mid–late	390.17	Precursor for GSH and phospholipids; substrate limitation
lysoPC a C16:1	lysoPC a C16:1	↑	Progressive	335.82	Membrane turnover; lyso‐lipid rise with phospholipase activity
Glutamine	Gln	Transient ↑ then ↓	Early transient then decline	314.07	Donor for GSH synthesis; falls as substrate pool shrinks
PC aa C34:2	PC aa C34:2	↓	Progressive	313.35	Membrane phospholipid loss with storage
PC aa C36:5	PC aa C36:5	↓	Progressive	302.34	Membrane phospholipid loss with storage
SM C18:1	SM C18:1	↓	Progressive	274.61	Sphingomyelin decline indicates membrane remodeling
PC aa C36:4	PC aa C36:4	↓	Progressive	273.57	Membrane phospholipid loss with storage
Lysine	Lys	↓/variable	Mid–late	272.66	Protein turnover/adsorption; storage stress
Creatinine	Creatinine	↑	Progressive	264.13	Metabolic by‐product accumulation
Ornithine	Orn	↑	Progressive	253.60	Urea‐cycle link; nitrogen handling

*Note*: Each entry reports the metabolite's common name and platform ID; its direction across storage (↑ increase, ↓ decrease, “variable” non‐monotonic); the approximate timing of the dominant change; the Hotelling's *T* ^2^ statistic from MEBA (larger values indicate stronger time‐dependent change); and a brief note on biological relevance. The values summarize storage‐related metabolic alterations consistent with storage lesions.

We additionally applied linear modeling with donor ID as a blocking factor and week 0 as the reference. This analysis revealed that 118 metabolites significantly changed their concentrations during storage (Data S1, worksheet IV). The top metabolites with increased concentrations in the supernatant of RBCs were Glycine (Gly), Octadecenoylcarnitine (C18:1), Octadecenoylcarnitine (C18:2), and Histidine (His). Conversely, the top metabolites with decreased concentrations were Methionine (Met), Hexoses (H1), Sphingomyelin C18:1 (SM C18:1), and Lysophosphatidylcholine a C16:1 (lysoPC a C16:1).

**FIGURE 3:**
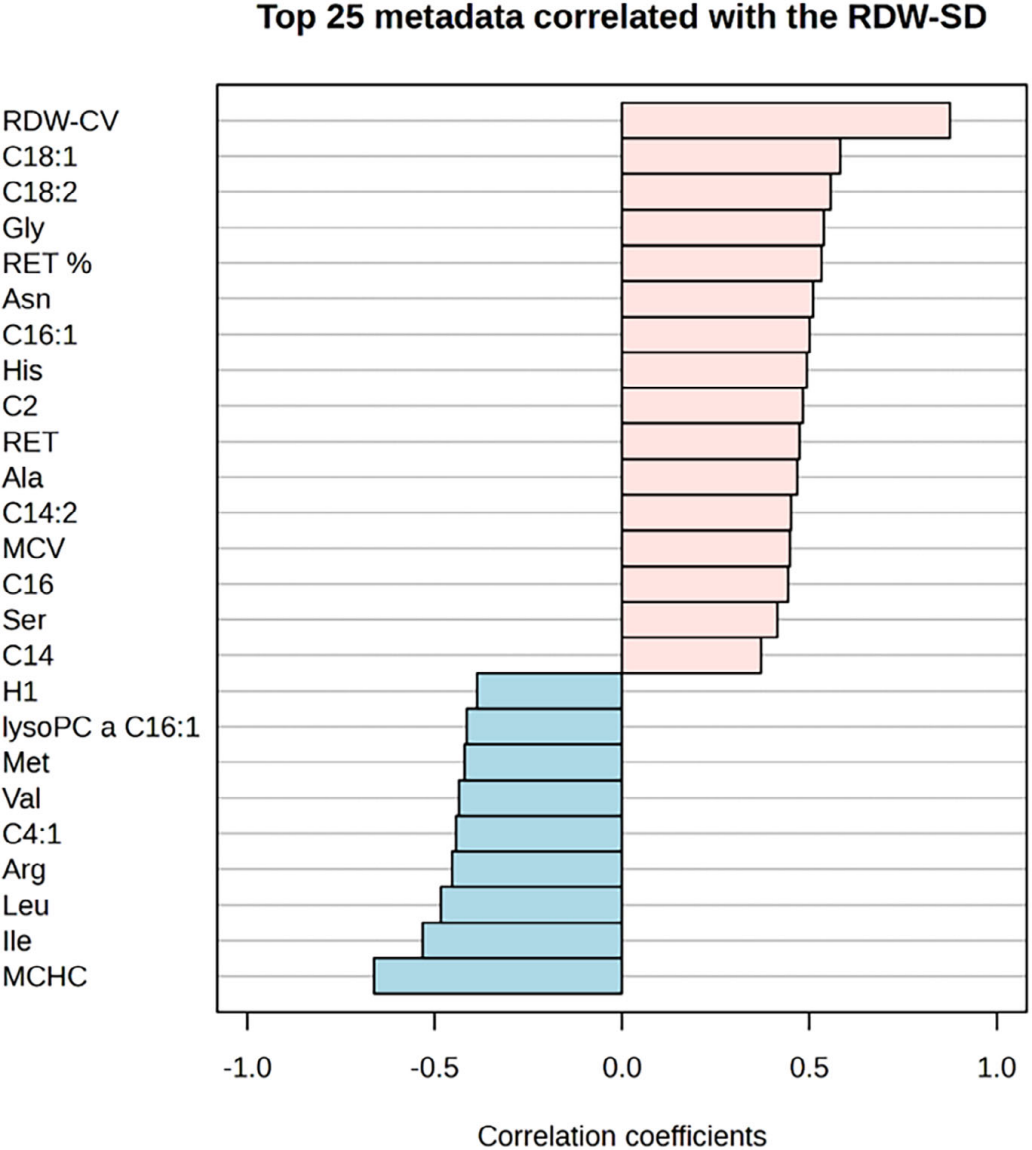
Top 25 metadata correlated with RDW‐SD in stored RBCs. The correlation between these metadata features and RDW‐SD can help identify key factors that influence red blood cell stability during storage. Metabolites with high positive correlations may be associated with storage‐induced damage, whereas metabolites with high negative correlations may be protective against such changes. This information is valuable for improving storage practices and ensuring the quality of stored packed RBCs for transfusions.

We conducted a 20‐parameter hemogram analysis using the Sysmex analyzer, which included reticulocyte differential parameters on pRBC samples over a 6‐week storage period. In addition, extHb was measured on supernatant of pRBC samples. Key parameters such as RDW‐SD (Red Cell Distribution Width—Standard Deviation), MCH (Mean Corpuscular Hemoglobin), MCV (Mean Corpuscular Volume), and MCHC (Mean Corpuscular Hemoglobin Concentration) were prioritized due to their stability and insensitivity to sample dilution required for Sysmex analyzer measurements (Data S1, worksheet I). These metrics are critical for assessing stored RBC integrity, detecting early hemolysis, and identifying storage‐related lesions. Notably, RDW‐SD, which quantifies variation in RBC size, showed increases indicative of cell fragmentation or storage‐induced damage. To investigate associations between these hemogram parameters and metabolic changes, we performed correlation analyses between RDW‐SD, extHb, and metabolomic/QM markers. The top 25 correlated metabolites and parameters are presented in Figure 3.

**FIGURE 4:**
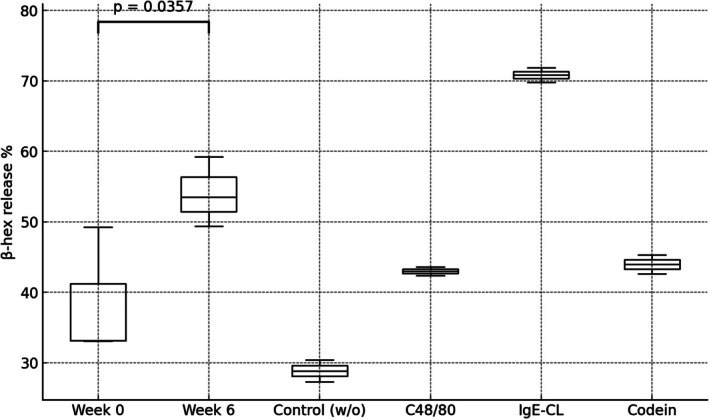
Comparison of degranulation in mast cell culture supplemented with supernatant from stored RBCs at Week 0 vs. Week 6. Mast cell cultures were supplemented with 10% (v/v) supernatant from packed RBCs collected at either Week 0 or Week 6. Mast cell degranulation was measured using a β‐hexosaminidase (ß‐hex) assay. The % total ß‐hex release from Week 0 versus Week 6 was compared using a paired *t*‐test. The boxplot shows the distribution of degranulation percentages for Week 0, Week 6, and additional conditions (IgE‐CL, C48/80, and Codeine stimulation of non‐supplemented MCs as positive control for degranulation). The *p*‐value from the paired *t*‐test between Week 0 and Week 6 is indicated on the plot.

To investigate the functional consequences of transfusing pRBCs with differing storage times, we compared fresh (Week 0) units and extended‐storage (Week 6) units for their effects on mast cell degranulation. Mast cell cultures were supplemented with 10% supernatant derived from either 6‐week‐old RBC units or fresh RBC units (stored for only a few hours post‐processing). As shown in Figure 4, mast cell degranulation was significantly increased after supplementation with supernatant from 6‐week‐old pRBC units compared to fresh (Week 0) units (*p* = .036, paired *t*‐test).

## 4 DISCUSSION

This study provides a comprehensive evaluation of the effects of prolonged storage of RBCs in PAGGS‐M, and clarifies both metabolic changes and their potential immunological implications. Over the 42‐day storage, significant alterations were observed in key metabolite classes, including amino acids (e.g., methionine), acylcarnitines, phospholipids, and hexoses. Unsupervised PCA revealed three distinct phases of metabolic change: unique profiles for Weeks 0 and 1, followed by a period (Weeks 2–6) of relative stability with no further substantial alterations. These findings are consistent with results for SAGM‐stored RBCs, which also showed three metabolic phases defined as an initial period of high metabolic activity (Days 0–7), a transitional phase (Days 7–14), and a final phase after Day 14 characterized by a marked reduction in metabolic processes.^16^ Although the two studies measured different metabolites and used different additive solutions, the similar metabolic phases observed suggest that the overall storage‐related changes were not drastically different between the two solutions. Importantly, the comparison of RBCs stored in PAGGS‐M and SAGM showed no impact on post‐transfusion recovery (PTR) at either 2 or 35 days.^23^ However, a statistically significant difference in PTR was observed when comparing day 2 and day 35 for both additive solutions. This underscores the need to establish a storage duration threshold at which PTR changes may become clinically relevant. The pattern visualized by PCA analysis in Figure 1A warrants further investigation as a means to define this threshold.

Our findings highlight the critical need to identify reliable biomarkers that can accurately assess both the metabolic aging of stored RBCs and their exposure to oxidative stress during storage. A key finding from our data is the early and substantial depletion of methionine, which coincides with the transition into the critical third metabolic phase of storage. In most RBC units, methionine levels reached near‐depletion by the end of the second metabolic phase. This aligns with the work of Reisz^17^ and D'Alessandro et al.,^12^, ^24^ who demonstrated that methionine consumption is directly linked to storage duration and oxidative hemolysis.

Methionine's depletion is critical because it is central to erythrocyte proteostasis.^25^, ^26^ As the precursor of S‐adenosylmethionine (SAM), it sustains the cell's methylation potential, which in turn fuels the PIMT/PCMT1 enzyme system.^25^ This system is essential for repairing L‐isoaspartyl damage on long‐lived membrane and cytoskeletal proteins that RBCs cannot replace de novo.^27^ Consistent with this framework, oxidative stress promotes asparagine deamidation to aspartate and formation of methyl‐Asp in key structural and metabolic proteins (Band 3, hemoglobin, ankyrin, protein 4.1, β‐spectrin, aldolase, GAPDH, BPGM, LDH, catalase),^28^, ^29^, ^30^ indicating cumulative protein damage and repair pressure. Importantly, reversible methylation of cytoskeletal/membrane proteins such as Band 3 and ankyrin is well established in RBCs^27^, ^29^ and is fed by methionine‐derived SAM transmethylation,^17^ thereby supporting membrane integrity and limiting proteolysis during storage.

It is important to distinguish methionine's role in proteostasis from the GSH/GSSG antioxidant system. Although methionine could, in principle, supply cysteine via transsulfuration, mature RBCs lack this pathway^31^ and the requisite enzymes, cystathionine β‐synthase and cystathionine γ‐lyase, are not detected in the RBC proteome.^32^ Our unpublished data are consistent with this, as methionine supplementation did not increase cysteine concentrations. Consequently, cysteine and GSH in stored RBCs are maintained by substrate transport and γ‐glutamyl‐cycle salvage rather than by methionine.^16^ This salvage pathway also fails during storage, because intracellular amino‐acid substrates fall^31^ and transporter activity reduces due to ATP loss, placing cysteine, glutamate, and glycine at the operative bottlenecks for redox buffering.^33^

Our results align with a substrate‐limited model. Supernatant glycine (Gly) and glutamate (Glu) increased progressively, whereas glutamine (Gln) rose transiently and then declined. We summarized this with a GSH‐turnover index, Gly/(Glu + Gln), which increased monotonically throughout storage (Data S1, worksheet V). This rise indicates that export of used glutathione and its extracellular γ‐glutamyl cycling outpace the cell's diminishing substrate supply. Consistent with our model, Rolfsson et al. observed that 5‐oxoproline, a marker of γ‐glutamyl‐cycle dysfunction, accumulates progressively in both intra‐ and extracellular compartments during storage.^34^ Taken together, these trends show that stored RBCs depend on a substrate‐limited, salvage‐dominated GSH turnover, reducing the capacity to buffer oxidative stress in mid to late storage and further underscoring the importance of methionine‐supported proteostasis and redox buffering.

Altogether, early methionine loss weakens methylation‐based protein maintenance, and the concurrent decline of the principal GSH/GSSG defense further exacerbates cellular vulnerability. In this context, reformulating RBC storage additive solutions to preserve methionine and prevent its depletion, which is commonly observed with conventional additives,^12^, ^13^ could significantly improve the quality of stored RBCs. Evidence from in vivo studies further highlights methionine's essential role in one‐carbon metabolism, which supports antioxidant defenses and protein‐repair mechanisms that are critical for RBC function under oxidative stress.^24^, ^25^, ^35^, ^36^, ^37^

Another notable alteration during RBC storage is increased membrane rigidity, which progressively exacerbates anisocytosis. This structural alteration is effectively captured by RDW‐SD and RDW‐CV, which represent the absolute variability and the variability relative to MCV in RBC size distribution, respectively.^38^, ^39^ We demonstrate here that both RDW‐SD and RDW‐CV increase statistically significant and correlate with storage duration, reflecting progressive declines in RBC deformability and heightened anisocytosis in stored RBCs. Our finding is also supported by several studies investigating RDW and its connection to the RBC deformability in in vivo and in vitro.^38^, ^40^, ^41^, ^42^, ^43^

The rationale for correlating RDW‐SD with metabolic markers is based on the mechanistic link between metabolic exhaustion and erythrocyte morphological remodeling. Oxidative stress impairs cytoskeletal and membrane proteins, promoting membrane loss and progressive shape alterations in red blood cells. These processes increase heterogeneity in cell size, which is quantitatively reflected by RDW‐SD.^38^, ^44^, ^45^ Accordingly, we observed a strong link between metabolic alterations and membrane integrity during storage. Supernatant C18:1 (oleic acid) and C18:2 (linoleic acid) show statistically significant positive associations with RDW‐SD (Pearson *r* = .53 and .44, both *p* < .001). These relationships remain directionally consistent in mixed‐effects models adjusted for storage time with a random intercept for donor (Data S1, worksheet VI). In parallel, other acylcarnitines accumulate in the supernatant (e.g., C18, C18:1, C18:2, C16, C16:1, C16:2, C14), indicating shifts in membrane lipid composition, while phosphatidylcholines and sphingomyelins decrease, consistent with progressive membrane phospholipid loss and degradation. The loss of membrane fatty acids, critical for maintaining fluidity and permeability, highlights storage‐induced compromises to membrane integrity.^37^, ^46^, ^47^ Oxidative stress, driven by ROS and hemoglobin's redox activity, accelerates lipid peroxidation, releasing free fatty acids and increasing acylcarnitine in the supernatant.^48^ These bioactive lipids, particularly C18 acylcarnitine, are known to activate mast cells, promoting histamine release and intracellular Ca^2+^ mobilization,^49^ potentially triggering allergic transfusion reactions (ATRs).

ATRs, which affect up to 2% of transfusions involving RBCs, platelets, or plasma^50^, ^51^ are influenced by multiple factors, including donor, product, and recipient characteristics. Among these, RBC storage duration is increasingly recognized as a key determinant of ATR risk.^52^, ^53^ In line with this, our study revealed significant accumulation of C18 acylcarnitine, a known contributor to mast cell activation, in RBC supernatants over extended storage. Considering the pivotal role of mast cells in ATRs,^51^, ^54^ we investigated how supernatants from different storage intervals affect mast cell degranulation. Our findings indicate that Week 6 supernatants induce markedly higher mast cell degranulation compared to Week 0, suggesting that duration of RBC storage cumulatively potentiates mast cell‐driven hypersensitivity reactions. Such transfusion‐driven mast cell hyperactivation may intersect with broader ATR pathways, potentially worsening acute lung injury and anaphylactoid reactions.^55^, ^56^, ^57^

## 5 STUDY LIMITATIONS

Our analyses prioritized cohort‐level storage‐time effects; donor‐level covariates (age, sex, ABO/Rh) were not formally modeled. Although the cohort was balanced on these variables, residual donor variation may persist. Functional validation was constrained by a small sample size and a single β‐hexosaminidase endpoint; future studies should incorporate orthogonal assays and larger cohorts. The targeted panel did not capture the full lipidome or redox intermediates, limiting pathway coverage. Findings reflect our processing conditions and additive solution, so generalizability to other workflows may be incomplete. As an observational study, residual confounding by unmeasured donor factors cannot be excluded. Finally, we did not assess post‐transfusion outcomes, so clinical implications remain inferential.

## 6 CONCLUSION

Using quantitative metabolomics, we investigated metabolic alterations in the supernatant of stored RBCs and their implications for transfusion safety. The supernatant, a mixture of plasma and additive solution, provides nutrients for RBCs during storage but gradually accumulates metabolic waste. This fluid is transfused into patients, making its composition clinically relevant. Our findings indicate that prolonged storage leads to increased levels of acylcarnitines, bioactive lipids associated with mast cell degranulation and potentially linked to allergic transfusion reactions. In parallel, we observed methionine depletion, underscoring its critical role in maintaining RBC membrane integrity and protecting against oxidative stress. A mast cell degranulation assay confirmed the functional impact of these metabolic changes, revealing heightened mast cell activation in correlation with longer RBC storage. These findings suggest that optimizing RBC storage solutions, such as through methionine supplementation, could help mitigate storage‐induced damage and improve transfusion outcomes. Further in vivo studies are needed to confirm these findings and develop tailored RBC concentrates for patient groups particularly susceptible to ATRs and other storage‐related adverse effects.

## AUTHOR CONTRIBUTIONS

Conceptualization: Gürkan Bal. Literature search: Gürkan Bal, Abdulgabar Salama, Maia Dzamashvili, Nidal Toman. Writing—original draft preparation: Gürkan Bal. Writing—review and editing: Gürkan Bal, Abdulgabar Salama, Nidal Toman, Maia Dzamashvili, Magda Babina. Experiments: Gürkan Bal, Zhuoran Li, and Maia Dzamashvili. Data analysis: Gürkan Bal, Nidal Toman. Visualization: Gürkan Bal and Nidal Toman. Funding acquisition: Gürkan Bal and Abdulgabar Salama. All authors have read and agreed to the published version of the manuscript.

## CONFLICT OF INTEREST STATEMENT

The authors have disclosed no conflicts of interest.

## Supporting information


**Data S1.** Supporting Information.

## Data Availability

The data that supports the findings of this study are available in the Supporting Information of this article.
